# A Cystine Transporter Mediates Nutrient Acquisition and Redox Balance During Wheat Stripe Rust Infection

**DOI:** 10.1111/mpp.70172

**Published:** 2025-11-12

**Authors:** Wanlu Duan, Yanfei Zhang, Shuohui Yang, Sifan Chen, Jiawen Yuan, Chaoran Zhang, Zhensheng Kang, Jing Zhao

**Affiliations:** ^1^ College of Plant Protection Northwest A&F University Yangling Shaanxi People's Republic of China; ^2^ College of Horticulture and Plant Protection Henan University of Science and Technology Luoyang Henan People's Republic of China; ^3^ State Key Laboratory for Crop Stress Resistance and High‐Efficiency Production Northwest A&F University Yangling Shaanxi People's Republic of China

**Keywords:** amino acid transporter, cysteine, cystine, hydrogen peroxide, redox, rust fungi

## Abstract

Amino acid uptake is crucial for the pathogenicity of *Puccinia striiformis* f. sp. *tritici* (Pst), the causative agent of wheat stripe rust. In this study, we investigated the dynamics of cystine accumulation in wheat leaves during Pst infection and identified Pst cystine transporters involved in this process. Amino acid profiling revealed a marked increase in cystine content at early infection stages. Phylogenetic analysis and expression profiling identified four candidate Pst cystine transporter genes, among which *PstAZ2B02G00053* (designated as *PstCYN1*) was functionally validated through yeast complementation assays. Subcellular localisation studies confirmed PstCYN1 as a plasma membrane transporter. Silencing of *PstCYN1* via BSMV‐VIGS and RNAi significantly reduced Pst virulence, as evidenced by decreased fungal biomass, reduced haustorial formation and fewer urediniospore pustules. Furthermore, apoplastic cystine accumulation and reactive oxygen species (ROS) levels were elevated in *PstCYN1*‐silenced plants, indicating that PstCYN1 mediates not only cystine uptake but also redox regulation at the infection interface. These findings highlight the critical role of *PstCYN1* in Pst nutrient acquisition and defence suppression, providing potential targets for enhancing wheat resistance against stripe rust.

## Graphical abstract

During wheat infection, *Puccinia striiformis* f. sp. *tritici* promotes cysteine export to the apoplast, where it is oxidised to cystine. The fungus then imports cystine via the PstCYN1 transporter to support its growth and quench host‐derived reactive oxygen species, thereby suppressing defence.

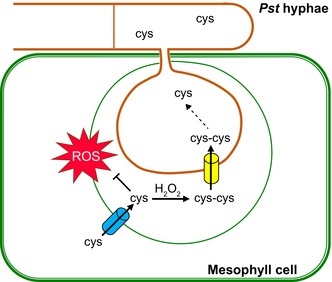

## 1 Introduction

Amino acids play pivotal roles in numerous biological processes throughout the life cycles of both fungi and plants. Beyond their fundamental role as protein constituents, amino acids serve as metabolic intermediates, signalling molecules and precursors for a wide array of crucial cellular components (Paudel et al. 2021). The flux of amino acid between intracellular compartments and various organs is regulated by amino acid transporters (AATs), which function not only as carriers but also as sensors of tissue nutrient status (Tegeder and Masclaux‐Daubresse 2018; Taylor 2014).

AATs in fungi are essential for nutrient acquisition, metabolic flexibility and environmental adaptation. These membrane‐bound transporters facilitate the uptake of extracellular amino acids, allowing fungi to supplement or bypass energetically costly de novo synthesis, which can require up to 75.5 ATP per amino acid (Wagner 2005). AATs are classified into several families, including the Amino Acid‐Polyamine‐Organocation (APC), Amino Acid/Auxin Permease (AAAP), Mitochondrial Carrier (MC), Lysosomal Cysteine Transporter (LCT) and Major Facilitator Superfamily (MFS) (Bianchi et al. 2019). In *Saccharomyces cerevisiae* , 24 AATs have been identified, such as the broad‐spectrum transporter GAP1 and Lyp1 (Donaton et al. 2003; Grenson et al. 1970; Karapanagioti et al. 2025). Functional specialisation is also evident in other fungi: *Aspergillus nidulans* uses PrnB for proline transport (Gournas et al. 2015), while *Cryptococcus neoformans* employs AAP4 and AAP5 to import branched‐chain amino acids and enhance stress tolerance (Martho et al. 2016). A recent study identified GNP2 as the primary proline transporter in *Candida albicans* , essential for proline‐induced filamentation, macrophage survival and oxidative stress resistance (Garbe et al. 2022). Deletion of GNP2 severely impaired these processes, underscoring the crucial role of amino acid transport in fungal pathogenicity. Collectively, these findings demonstrate that AATs are not only pivotal for nutrient acquisition but also integrate with regulatory pathways governing fungal development and host interaction (Nair and Sarma 2021; Garbe et al. 2022).

Sulphur is an essential macronutrient required for proper cellular function of all living organisms. In fungi, sulphur is acquired either in the form of inorganic sulphate or sulphur‐containing amino acids from the environment or their host (Linder 2018). Among these, cysteine plays a central role in sulphur metabolism, serving as a precursor for various sulphur‐containing cellular molecules (Moormann et al. 2025). However, due to the oxidative nature of the extracellular environment, free cysteine is highly unstable and rapidly oxidised to cystine. Most cells depend on cystine transporters to uptake cystine, which is subsequently reduced to cysteine in the cytosol through an NADPH‐dependent reaction (Koppula et al. 2021). Cystine transporters have been identified in bacteria, fungi and mammalian cells (Yadav and Bachhawat 2011; Banjac et al. 2008; Chonoles Imlay et al. 2015). In mammals, transporters such as the heterodimeric amino acid transporter b0, +AT (SLC7A9), the cystine‐glutamate antiporter xCT (SLC7A11) and AGT‐1 (SLC7A13) play key roles in redox homeostasis and are implicated in various disorders including cancer and cystinuria (Nagamori et al. 2016; Sleire et al. 2015; Pereira et al. 2015; Banjac et al. 2008). In unicellular eukaryotes, functional cystine transporters have been definitively demonstrated in pathogenic yeasts, but they are absent in nonpathogenic counterparts, suggesting a possible link between cystine transport and host–pathogen interactions (Yadav and Bachhawat 2011). Despite their importance, the functional characterisation of cystine transporters in obligate biotrophic fungi remains largely unexplored.

As strict obligate biotrophs, rust fungi rely on carbon and nitrogen transporters to acquire nutrients from host cells, with sucrose and amino acid transporters being particularly important. Some studies have highlighted that pathogens can develop strategies to compete with their hosts for sucrose. In *Uromyces fabae* , the hexose transporter HXT1 is localised in haustoria and facilitates the uptake of glucose and fructose (Voegele et al. 2001). Wheat stripe rust, caused by *Puccinia striiformis* f. sp. *tritici* (Pst), is one of the most devastating diseases of wheat, posing a serious threat to global wheat production and food security (Chen et al. 2014). Recent work on PsHXT1 provided the first in vivo evidence that sugar starvation restricts both pathogen growth and virulence (Chang et al. 2020). Although many amino acid transporter gene sequences have been reported in rust fungi, their functions remain largely uncharacterised (Struck 2015).

In this study, we identified and functionally characterised the cystine transporter PstCYN1 from Pst. PstCYN1 is induced during infection and localises to the plasma membrane, where it facilitates cystine uptake and contributes to pathogen growth while suppressing host immunity. Our findings demonstrate that PstCYN1 plays a crucial role in Pst pathogenicity. To our knowledge, this study is the first to directly reveal the significance of a fungal amino acid transporter in rust pathogenicity.

## 2 Results

### 2.1 Cystine Accumulation in Wheat Leaves During Early Pst Infection

**FIGURE 1:**
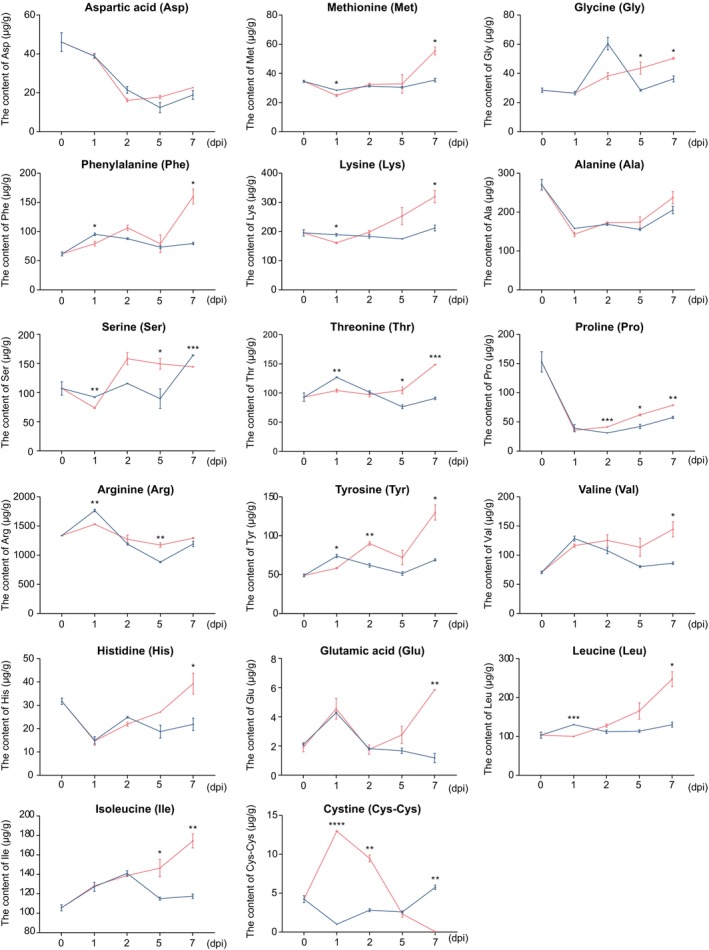
Temporal changes in amino acid profiles of wheat leaves following *Puccinia striiformis* f. sp. *tritici* (Pst) infection. Quantitative analysis of 17 amino acids in leaves from wheat seedling either mock‐inoculated (blue) or infected with Pst (red). Samples were collected at 0, 1, 2, 5 and 7 days post‐inoculation (dpi). Values represent the mean ± SD from three biological replicates (*n* = 3). Asterisks indicate statistically significant differences compared to samples at 0 dpi using two‐sided Student's *t* test (*p < 0.05, **p < 0.01, ***p < 0.001, ****p < 0.0001).

To investigate changes in amino acid levels in wheat upon Pst infection, we quantified the contents of 17 amino acids in wheat leaves at 0, 1, 2, 5 and 7 days post‐inoculation (dpi). Amino acid levels in Pst‐infected plants (CYR31) were compared with those in mock‐inoculated plants (water). The results revealed that most amino acids exhibited varying degrees of change in response to infection, suggesting that Pst infection profoundly affects amino acid biosynthesis or transport in wheat. Notably, cystine exhibited a particularly dramatic increase at the early stages of infection (1 and 2 dpi), with levels rising 12.0‐fold and 3.6‐fold, respectively (Figure 1). Given this remarkable change, we focused on the role of cystine in the wheat–Pst interaction in the subsequent analyses.

### 2.2 Identification and Expression Profiling of Four Candidate Cystine Transporter Genes in Pst

**FIGURE 2:**
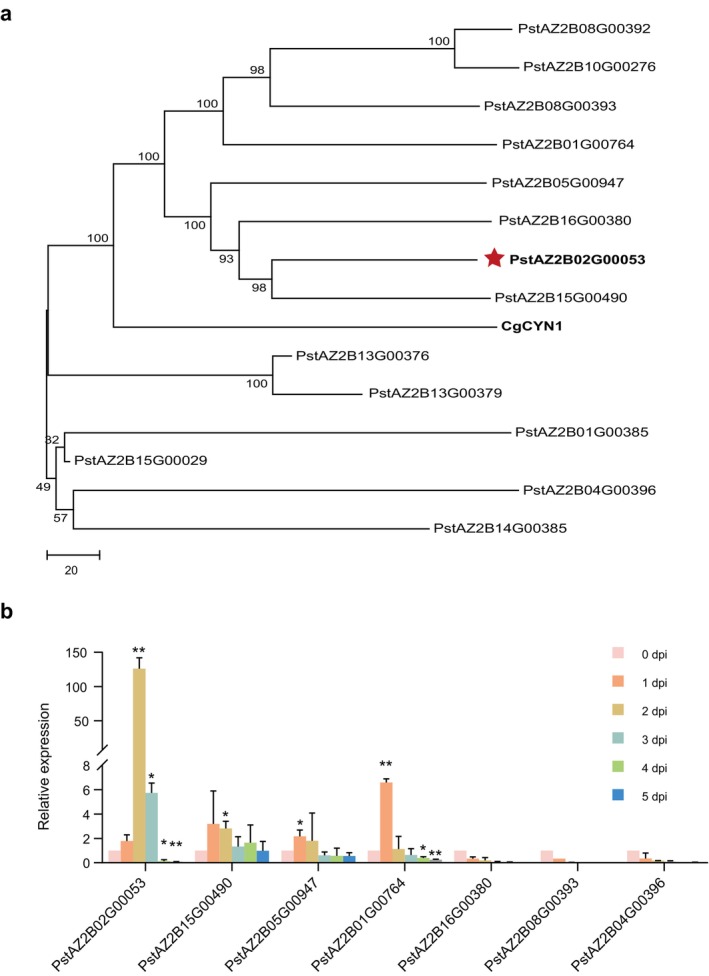
Phylogenetic relationship and expression dynamics of putative cystine transporter genes in *Puccinia striiformis* f. sp. *tritici* (Pst) genome. (a) Neighbour‐joining phylogenetic tree showing the relationship between the known cystine transporter CgCYN1 from *Candida glabrata* and homologous proteins identified in the Pst genome. The tree was constructed using MEGA7 software with 1000 bootstrap replicates; branch lengths indicate evolutionary distance and bootstrap support is shown at each node. (b) Temporal expression profiles of Pst candidate cystine transporter genes during infection. Transcript levels were measured at 0, 1, 2, 3, 4 and 5 days post‐inoculation (dpi) using the 2^−ΔΔ*Ct*^ method, with normalisation to *PstEF1*. Data represent mean values from three biological replicates ± SD. Asterisks denote statistically significant differences relative to 0 dpi (two‐sided Student's *t* test; *p < 0.05, **p < 0.01).

Previous studies have shown that numerous amino acid transporter genes in Pst are differentially expressed during wheat infection, suggesting their involvement in the wheat–Pst interaction (Zhao et al. 2021). To identify potential cystine transporters in Pst, the *Candida glabrata* cystine transporter gene *CgCYN1* (Yadav and Bachhawat 2011) was used as a query to search the Pst AZ‐2 genome (Wang et al. 2024). Based on phylogenetic analysis, seven candidate genes with homology to *CgCYN1* were identified (Figure 2a). Reverse transcription‐quantitative PCR (RT‐qPCR) analysis revealed that *PstAZ2B02G00053*, *PstAZ2B02G00490*, *PstAZ2B02G00947* and *PstAZ2B02G00764* were strongly induced during the early infection stages (Figure 2b). These findings suggest that these four genes may function as cystine transporters involved in early Pst development during host infection.

### 2.3 Functional Complementation in Yeast Demonstrates PstCYN1 as a Cystine Transporter

**FIGURE 3:**
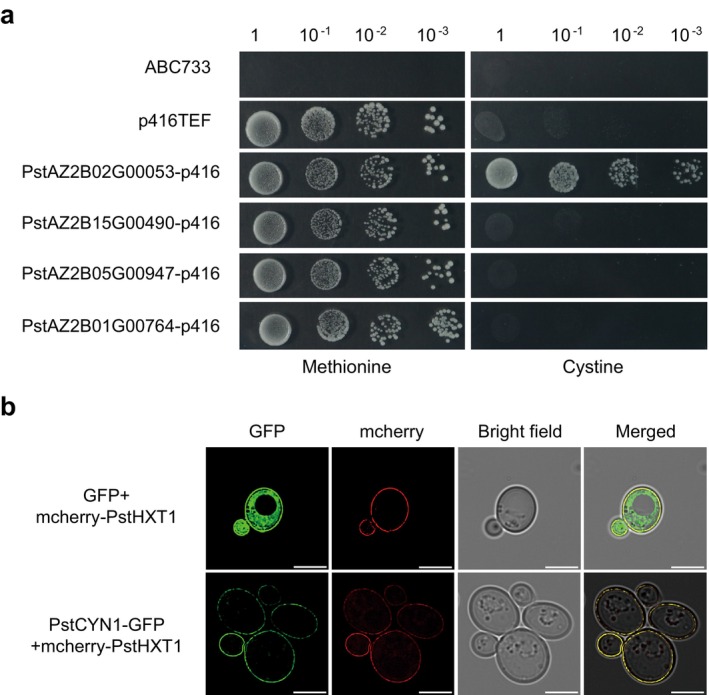
Functional characterisation and subcellular localisation of PstCYN1. (a) Functional complementation assay of candidate *Puccinia striiformis* f. sp. *tritici* (Pst) cystine transporter genes in *Saccharomyces cerevisiae* *met15Δ* mutant strain ABC733. Yeast cells were transformed with constructs encoding PstAZ2B02G00053, PstAZ2B15G00490, PstAZ2B05G00947, and PstAZ2B01G00764 cloned into the p416TEF vector, along with an empty vector (EV) control. Transformants were precultured in methionine‐supplemented minimal medium, serially diluted, and spotted onto selective medium containing cystine as the sole sulphur source to assess growth restoration. (b) Subcellular localisation of PstCYN1 in yeast. mcherry‐PstHXT1 was used as a plasma membrane marker. A GFP‐tagged version of PstAZ2B02G00053 (GFP‐PstCYN1) or the GFP‐only control was co‐expressed with mcherry‐PstHXT1 in yeast ABC733 cells. Fluorescence signals were observed at 2 days post‐transformation using fluorescence microscopy. Images include GFP fluorescence (left), bright‐field (middle), and merged views (right). Scale bars: 5 μm.

The *S. cerevisiae* *met15*Δ strains are organic sulphur auxotrophs that can metabolise cysteine and methionine but cannot grow on cystine (for strain ABC733) or cysteine (for strain ABC1904) because they lack efficient transporters to uptake these compounds from the medium (Thomas and Surdin‐Kerjan 1997; Yadav and Bachhawat 2011). Previous studies have shown that heterologous expression of a plasma membrane‐localised cystine transporter enables growth on cystine (Yadav and Bachhawat 2011). We employed this system to evaluate whether the four candidate Pst cystine transporter genes can confer cystine uptake ability in yeast. The constructs p416TEF‐*PstAZ2B02G00053*, p416TEF‐*PstAZ2B02G00490*, p416TEF‐*PstAZ2B02G00947*, p416TEF‐*PstAZ2B02G00764* and the empty vector control (p416TEF) were transformed into *S. cerevisiae* ABC733 cells. Transformants were precultured in minimal medium supplemented with methionine and other nutrients overnight, then tested for growth on cystine as the sole sulphur source. Only yeast expressing *PstAZ2B02G00053* was able to grow on cystine, indicating that this gene encodes a functional cystine transporter (Figure 3a). In addition, yeast strain ABC1904 expressing *PstAZ2B02G00053* failed to grow on medium with cysteine as the sole sulphur source, suggesting that PstAZ2B02G00053 specifically mediates cystine, but not cysteine, transport (Figure S1). Collectively, these results demonstrate that *PstAZ2B02G00053* functions as a cystine‐specific transporter in Pst. We therefore designated this gene as Pst cystine transporter 1 (*PstCYN1*).

### 2.4 PstCYN1 Localises to the Cell Membrane in Yeast and *Nicotiana* *benthamiana*

Further analysis of PstCYN1 revealed that it possesses typical characteristics of the amino acid–polyamine–organocation (APC) superfamily, including 12 predicted transmembrane domains (Figure S2). To determine its subcellular localisation, a PstCYN1‐GFP fusion protein was constructed and expressed in *S. cerevisiae* . Fluorescence microscopy showed that the GFP signal overlapped with the red fluorescence signal of the plasma membrane marker mcherry‐PstHXT1 (Chang et al. 2020), confirming the membrane localisation of PstCYN1 in yeast (Figure 3b). Transient expression of PstCYN1‐GFP in *N. benthamiana* leaves further confirmed its localisation to the plasma membrane (Figure S3).

### 2.5 Silencing of *PstCYN1* Reduces Pst Pathogenicity in Wheat

To investigate the function of *PstCYN1* during the interaction between wheat and Pst, specific silencing fragments of *PstCYN1* were used to construct a barley stripe mosaic virus (BSMV)‐based virus‐induced gene silencing (VIGS) vector (Figure S4a). Obvious photobleaching in BSMV:*TaPDS*‐inoculated plants confirmed that the VIGS system functioned effectively in the wheat. At 10 days post‐BSMV infection, the second leaves of wheat inoculated with BSMV:γ (control) and BSMV:*PstCYN1* displayed similar mild chlorotic mosaic symptoms on the fourth leaves (Figure S4b). The fourth leaves were then inoculated with virulent Pst race CYR31. At 14 dpi, wheat leaves silenced for *PstCYN1* showed a significant reduction in urediniospore production compared to the control (Figure S4b). RT‐qPCR confirmed effective gene silencing: *PstCYN1* transcript levels in BSMV:*PstCYN1*‐infected leaves were reduced by approximately 62% at 1 dpi and 87% at 2 dpi compared to BSMV:γ‐inoculated plants (Figure S4c). Correspondingly, the relative fungal biomass was significantly reduced in *PstCYN1*‐silenced plants (Figure S4d). Histological analyses revealed significant reductions in hyphal length, number of infection hyphae, haustorial mother cells and haustoria at 1 and 2 dpi, as well as a decrease in infection area at 5 dpi (Figure S4e–i).

**FIGURE 4:**
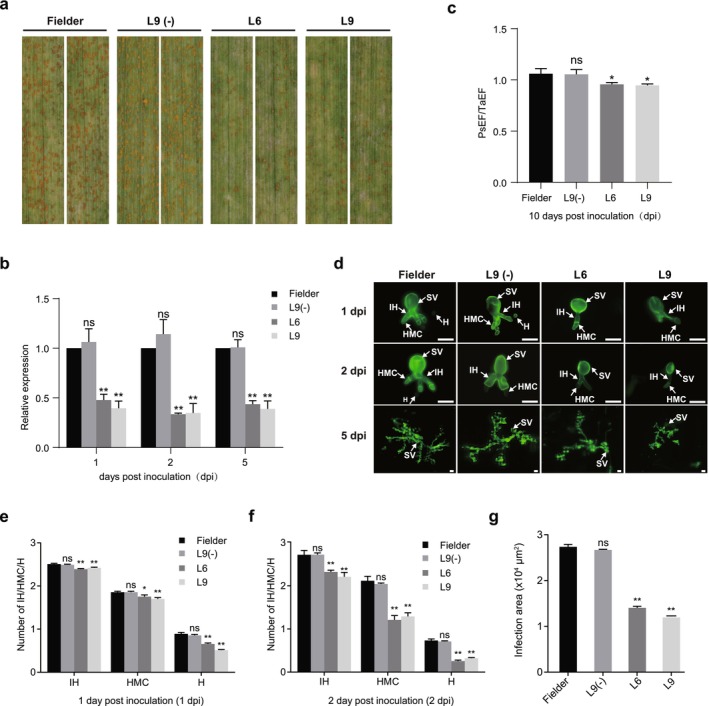
Host‐induced gene silencing (HIGS) of *PstCYN1* attenuates the virulence of *Puccinia striiformis* f. sp. *tritici* (Pst). (a) Disease phenotypes of *PstCYN1*‐silenced wheat lines (L6 and L9) and a non‐silenced segregant line (L9−) following inoculation with Pst race CYR31. Leaf symptoms were documented at 14 days post‐inoculation (dpi). (b) Relative transcript levels of *PstCYN1* in RNAi transgenic lines compared to wild‐type (WT) wheat at 1, 2 and 5 dpi, as determined by reverse transcription‐quantitative PCR. (c) Quantification of fungal biomass in inoculated leaves at 10 dpi. Biomass was estimated by normalising Pst genomic DNA (*PstEF1*) to host DNA (*TaEF‐1α*). (d) Microscopic visualisation of Pst infection structures in wheat leaves at 1, 2 and 5 dpi. Samples were stained with WGA‐488 to highlight fungal components. H, haustorium; HMC, haustorial mother cell; IH, infection hypha; SV, substomatal vesicle. Scale bars: 20 μm (1 and 2 dpi), 200 μm (5 dpi). (e, f) Quantitative analysis of Pst infection structures at 1 dpi (e) and 2 dpi (f), including the average number of IH, HMC, and H per infection site. (g) Measurement of infection site area in wheat leaves at 5 dpi. Data represent mean values from three biological replicates ± SD. Asterisks denote statistically significant differences relative to 0 dpi (two‐sided Student's *t* test; *p < 0.05, **p < 0.01).

To further validate the virulence function of *PstCYN1*, we generated *PstCYN1*‐RNAi transgenic wheat lines. Two positive RNAi lines (L6 and L9) and one negative RNAi segregant line (L9‐) were selected for resistance evaluation (Figure S5). Consistent with the VIGS results, the positive RNAi lines displayed significantly enhanced resistance, characterised by reduced urediniospore pustule formation (Figure 4a). The transcription levels of *PstCYN1* were substantially repressed at 1, 2 and 5 dpi in positive RNAi lines (Figure 4b). Moreover, fungal biomass was markedly decreased in the positive RNAi plants (Figure 4c), consistent with the reduced numbers of infection hyphae, haustoria mother cells and haustoria at early time points, and smaller infection areas at 5 dpi (Figure 4d–g).

### 2.6 Impaired Cystine Transport in *PstCYN1* ‐Silenced Plants Lead to Hydrogen Peroxide Accumulation

**FIGURE 5:**
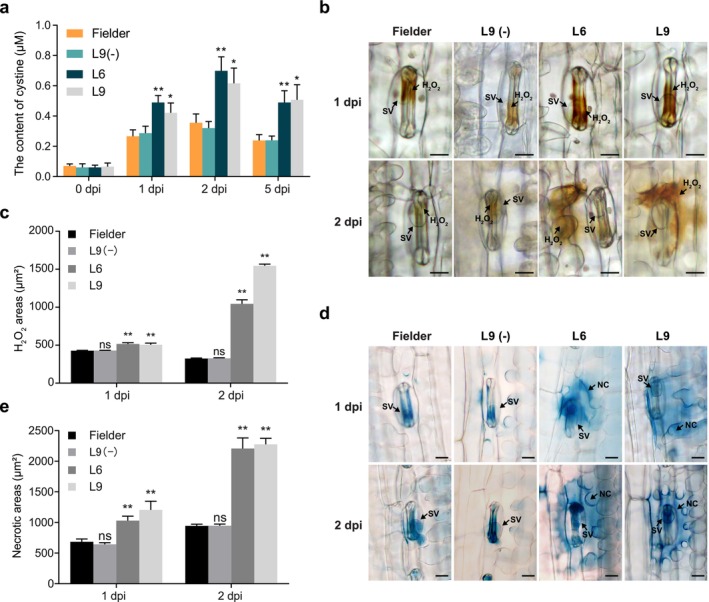
Silencing of *PstCYN1* reduces apoplastic cystine contents and promotes hydrogen peroxide (H_2_O_2_) at infection sites. (a) Quantification of cystine levels in the apoplastic fluid of wheat leaves infected with *Puccinia striiformis* f. sp. *tritici* (Pst). Data represent mean values ± SD from three biological replicates. Asterisks denote statistically significant differences compared to wild‐type Fielder at each time point (two‐sided Student's *t* test; *p < 0.05, **p < 0.01). (b) Histochemical detection of H_2_O_2_ accumulation at infection sites in wheat leaves at 1 and 2 days post‐inoculation (dpi). SV, substomatal vesicle. Scale bars: 20 μm. (c) Quantification of H_2_O_2_‐positive areas at infection sites at 1 and 2 dpi. Values are expressed as means ± SD (*n* = 3). Asterisks indicate statistically significant differences relative to wild‐type Fielder (two‐sided Student's *t* test; **p < 0.01; ns, not significant). (d) Histochemical staining of necrotic cell death at Pst infection sites in transgenic RNAi lines at 1 and 2 dpi. Trypan blue staining was used to visualise dead cells. NC, necrotic cell death; SV, substomatal vesicle. Scale bars: 20 μm. (e) Quantification of necrotic area at infection sites at 1 and 2 dpi. Data are presented as means ± SD from three biological replicates. Statistical differences were evaluated using a two‐sided Student's *t* test (**p < 0.01; ns, not significant).

To investigate the role of the Pst cystine transporter during infection, we quantified cystine levels in the apoplast of *PstCYN1*‐silenced plants and wild‐type plants at different time points. As expected, apoplastic cystine levels increased markedly during infection in both plant types (Figure 5a). However, *PstCYN1*‐silenced plants consistently showed higher levels of apoplastic cystine compared to wild‐type plants, suggesting that silencing of *PstCYN1* impairs the transport of cystine from the host apoplast into the pathogen.

Given that cystine is the oxidised form of cysteine, it is likely generated by the oxidation of apoplastic cysteine via hydrogen peroxide produced as part of the wheat defence response. Based on this, we hypothesize that the Pst cystine transporter not only absorbs cystine from the apoplast as a nutrient but also reduces the oxidative state at the pathogen–host interface by consuming hydrogen peroxide through cystine transport. To test this hypothesis, we examined the accumulation of reactive oxygen species (ROS) at Pst infection sites. ROS levels were significantly higher in *PstCYN1*‐silenced plants (lines L6 and L9) compared to wild‐type and transgenic negative control plants (L9[−]), as confirmed by quantification of ROS‐stained areas (Figure 5b,c). Additionally, cell necrosis was significantly increased in the silenced plants (Figure 5d,e). These findings demonstrate that *PstCYN1* plays a crucial role in regulating the redox balance at the wheat–Pst interaction site by facilitating cystine transport from the apoplast into the pathogen. Impairment of this transporter leads to the accumulation of both cystine and hydrogen peroxide, intensifying host oxidative defences and cell necrosis. This suggests that *PstCYN1* is essential not only for nutrient acquisition but also for modulating the oxidative environment at the infection interface, thereby contributing to Pst pathogenicity.

## 3 Discussion

**FIGURE 6:**
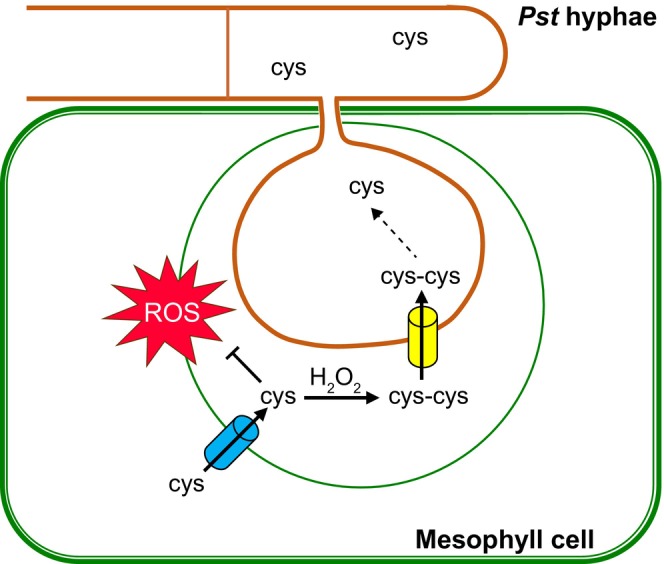
A working model for PstCYN1 promoting *Puccinia striiformis* f. sp. *tritici* (Pst) infection. During infection of wheat by Pst, the pathogen manipulates host amino acid transporters to export cysteine into the extrahaustorial matrix, where it is oxidised by host‐derived reactive oxygen species (ROS) into cystine. PstCYN1, a transporter highly expressed and localised on the fungal plasma membrane, rapidly imports cystine into the fungal hyphae, where it is subsequently reduced back to cysteine. This PstCYN1‐mediated cystine uptake serves dual functions: It provides a nutritional source to support fungal growth and proliferation, and promotes the depletion of ROS by facilitating the conversion of cysteine to cystine, thereby suppressing host immune responses and enhancing pathogen colonisation and reproduction.

Amino acid uptake is a critical factor underlying the pathogenic success of biotrophic fungal pathogens during plant infection. In this study, we identified and functionally characterised PstCYN1 as a cystine transporter that plays a dual role in nutrient acquisition and suppression of host defences in stripe rust fungi (Figure 6). Our findings reveal that PstCYN1‐mediated cystine uptake is essential for fungal growth, infection structure development and modulation of the redox environment at the infection interface, highlighting its importance in Pst pathogenicity.

Our amino acid profiling revealed a striking accumulation of cystine in wheat leaves during the early stages of Pst infection, suggesting that apoplastic cystine availability increases shortly after pathogen entry and provides a nutrient source for Pst establishment. This accumulation likely results from pathogen‐induced export of cysteine via host amino acid transporters, followed by its rapid oxidation by host‐derived ROS. Several plant AATs are induced upon pathogen infection. For example, *Arabidopsis* amino acid transporter gene *AtLHT1* transcript levels rise during infection by the biotrophic fungus *Erysiphe cichoracearum* (Liu et al. 2010) and rice *OsLHT1* is up‐regulated upon inoculation with *Magnaporthe oryzae* (Guo et al. 2023). *Arabidopsis* infected by the necrotrophic fungus *Botrytis cinerea* increases UmamiT20 mRNA accumulation (Prior et al. 2024), while *Pseudomonas syringae* infection leads to an elevation of *AtCAT1* expression (Yang et al. 2024). These findings demonstrate how pathogens reprogramme plant amino acid transporters to promote their growth or modulate host defence (Tünnermann et al. 2022).

Although fungal cells can synthesise amino acids de novo, they also actively import amino acids via plasma membrane‐localised AATs from the extracellular environment. Despite their significance, relatively few fungal AATs have been functionally characterised, mainly in *S. cerevisiae* , *C. albicans* and *A. nidulans* (Yang et al. 2024). In plant‐pathogenic fungi, only three *U. fabae* AAT genes (*AAT1*, *AAT2*, *AAT3*) encoding YAT family permeases have been identified, each with distinct expression patterns (Struck 2015). AAT1 and AAT3 have a broad substrate spectrum, whereas AAT2 is haustorium‐specific with unknown substrate specificity; their roles in fungal growth or pathogenicity also remain unclear. Through phylogenetic, expression and functional analyses, we identified PstCYN1 as a bona fide cystine transporter. Silencing of *PstCYN1* significantly compromised Pst pathogenicity, as evidenced by reduced fungal biomass, impaired haustorial formation and decreased urediniospore production. Cysteine is a key precursor for sulphur‐containing molecules, including Fe‐S clusters, vitamins and enzyme cofactors. In fungal pathogens, cysteine is especially important for the synthesis of effector proteins, such as small secreted cysteine‐rich proteins (Nie et al. 2019; Pendleton et al. 2014; Wang et al. 2020; Kamoun 2007). These findings provide the first evidence that AATs in plant‐pathogenic fungi contribute directly to virulence and highlight the essential role of cystine uptake in Pst proliferation and reproduction during infection.

Notably, our data provide strong evidence that PstCYN1 contributes not only to nutrient uptake but also to redox regulation at the host–pathogen interface. Silencing of *PstCYN1* resulted in elevated apoplastic cystine and ROS accumulation, as well as increased host cell death, suggesting that PstCYN1‐mediated cystine import depletes apoplastic cystine, indirectly mitigating ROS levels and suppressing oxidative defence responses. Such a dual role in nutrient acquisition and immune suppression reflects Pst's sophisticated adaptation to the hostile plant apoplast, allowing the pathogen to balance nutrient uptake with evasion of plant immunity. Pathogens must manage host‐derived oxidative stress to establish infection. For example, in *Fusarium graminearum*, the transcription factor FgbZIP007 regulates glutathione biosynthesis to counter oxidative stress (Park et al. 2024). Similar redox‐modulating strategies have been described for cancer cells. Overexpression of cystine transporter genes in BL cells led to an enhanced resistance to oxidative stress by driving a highly efficient cystine/cysteine redox cycle without increasing intracellular GSH levels (Banjac et al. 2008; Koppula et al. 2021). This cystine/cysteine redox cycle may represent a shared strategy for oxidative stress tolerance in both fungal and animal cells. Interestingly, recent studies demonstrate cytosolic cysteine acts as a metabolic signal in plant immunity, reflecting plant responses to pathogen‐triggered cysteine synthesis and transport (Moormann et al. 2025).

Finally, while this study provides significant insights, several questions remain. Direct quantification of cystine flux at the infection site, characterisation of PstCYN1 transport kinetics and identification of other transporters or host factors involved in cystine dynamics will further refine our understanding of Pst nutrient acquisition strategies. In addition, structural studies of PstCYN1 may inform the design of specific inhibitors to block its activity.

In conclusion, we propose a model in which PstCYN1 functions at the frontline of the wheat–Pst interface to facilitate cystine uptake, supporting both the nutritional and redox requirements of Pst during infection. This dual functionality is critical for Pst virulence and highlights the sophisticated interplay between nutrient acquisition and immune suppression in the evolution of obligate biotrophy.

## 4 Experimental Procedures

### 4.1 Plant Materials and Strains

Wheat ( *Triticum aestivum* ) cultivar Suwon11 (Su11) and Fielder, as well as *N. benthamiana*, were used in this study. Both Su11 and Fielder are highly susceptible to Pst race CYR31. Wheat was grown in a growth chamber under controlled conditions: 16°C, 75% relative humidity and a photoperiod of 16 h light/8 h dark. *N. benthamiana* plants were grown under controlled conditions: 21°C, 75% relative humidity and a photoperiod of 16 h light/8 h dark. For Pst inoculation, plants were maintained in complete darkness at 12°C and 100% relative humidity for 24 h post‐inoculation and then returned to the standard growth conditions described above.

### 4.2 High‐Throughput Analysis of 17 Amino Acid in Wheat Leaves Infected by Pst

To quantify infection‐induced changes in amino acid composition, 0.1 g of wheat leaf tissue was collected at 0, 1, 2, 5 and 7 dpi. Samples were homogenised in 1.5 mL of 6 M hydrochloric acid containing 0.1% phenol to form a uniform paste. The homogenate was transferred to a 1.5 mL microcentrifuge tube and hydrolysed at 100°C for 20 h. After cooling to room temperature, 1 mL of the hydrolysate was concentrated to near dryness using a thermovap sample concentrator. The residue was resuspended in 1 mL of 0.1 M HCl and filtered through a 0.22 μm membrane filter.

For derivatisation, 200 μL of the filtered sample or amino acid standard solution was transferred to 1.5 mL microcentrifuge tubes. To each tube, 20 μL of L‐leucine internal standard solution was added, followed by 100 μL of triethylamine‐acetonitrile solution and 100 μL of isothiocyanate benzyl ester acetonitrile solution. The mixtures were vortexed thoroughly and incubated at 25°C for 1 h. Subsequently, 400 μL of hexane was added, followed by vigorous vortexing for 30 s and standing at room temperature for 10 min. The lower phase was collected, diluted 5‐fold with deionised water and filtered through a 0.45 μm needle filter.

Quantification was performed using high‐performance liquid chromatography (HPLC) on a Rigol L3000 system equipped with a Kromasil C18 reverse‐phase column (250 mm × 4.6 mm, 5 μm). Solvent A consisted of 7.6 g of anhydrous sodium acetate dissolved in 925 mL of water, adjusted to pH 6.5 with glacial acetic acid and supplemented with 70 mL of acetonitrile. The solution was filtered through a 0.45 μm membrane. Solvent B was 80% aqueous acetonitrile. The gradient elution programme was as follows: 0–2 min, 100% A; 2–15 min, 0%–10% B (linear); 15–30 min, 10%–30% B (linear); 30–33 min, 30%–45% B (linear); 33–38 min, 100% B; 38–45 min, 100% A. The flow rate was 1.0 mL/min, and the column temperature was maintained at 40°C. Sample analysis was conducted by Suzhou Comin Biotechnology Co. Ltd.

### 4.3 RNA Extraction and RT‐qPCR Analysis

To analyse the transcript levels of candidate amino acid transporter (AAT) genes, leaves from wheat cultivar Su11 inoculated with Pst race CYR31 were collected at 0, 1, 2, 3 and 5 dpi. Total RNA was extracted using the Quick RNA Isolation Kit (Huayueyang Biotechnology) following the manufacturer's protocol. First‐strand cDNA was synthesised from 2 μg of total RNA using the RevertAid First Strand cDNA Synthesis Kit (Thermo Scientific) according to the manufacturer's instructions.

RT‐qPCR was performed using gene‐specific primer pairs (Table S1) and ChamQ SYBR qPCR Master Mix (Vazyme) on a CFX Connect Real‐Time PCR Detection System (Bio‐Rad). Relative gene expression levels were calculated using the comparative 2^−ΔΔ*C*t^ method, with normalisation to the *PstEF1* reference gene. Each sample consisted of three biological replicates, with each replicate comprising pooled leaf tissue from three individual plants. Technical triplicates were included for each RT‐qPCR. Statistical significance was determined using an unpaired two‐tailed Student's *t* test.

### 4.4 Cystine Transport Activity Assay in *S. cerevisiae*

The full‐length coding sequences (CDS) of candidate AAT genes were amplified and cloned into the BamHI and XhoI restriction sites of the yeast expression vector p416TEF, resulting in constructs p416TEF‐*PstAZ2A02G00053*, p416TEF‐*PstAZ2A15G00490*, p416TEF‐*PstAZ2A05G00947* and p416TEF‐*PstAZ2A02G00764*. These recombinant plasmids were introduced into either yeast strain ABC733 ( *S. cerevisiae* MATa his3Δ1 leu2Δ0 met15Δ0 ura3Δ0) or ABC1904 ( *S. cerevisiae* MATa his3Δ1 leu2Δ0 met15Δ0 ura3Δyct1::HIS4). Strain ABC733 is unable to utilise cystine as a sole sulphur source, while ABC1904 is deficient in utilising cysteine as a sole sulphur source (Yadav and Bachhawat 2011). Yeast transformations were performed using a modified lithium acetate method as previously described (Gietz 2014).

For the dilution spotting assay, transformed yeast strains were cultured overnight in minimal medium supplemented with nutrients but lacking uracil. Cultures were diluted to an optical density at 600 nm (OD_600_) of 0.1 in fresh medium and incubated for an additional 6 h. Cells in the exponential growth phase were harvested, washed with sterile water, and resuspended to an OD_600_ of 0.2. Serial 10‐fold dilutions (1:10, 1:100 and 1:1000) were prepared, and 10 μL of each dilution was spotted onto the appropriate selective plates. Plates were incubated at 30°C for 2 days, and growth was documented by photography.

### 4.5 Subcellular Localisation of PstCYN1 in Yeast and *N. benthamiana*

For subcellular localisation analysis in yeast, the CDS of free eGFP, eGFP‐fused *PstCYN1* and mcherry‐fused *PstHXT1* were amplified using gene‐specific primers (Table S1) and inserted into the XhoI and BamHI sites of the pDR195 vector, generating pDR195‐eGFP, pDR195‐*PstCYN1*‐eGFP and PDR195‐mcherry‐*PstHXT1* respectively. Then the cell membrane marker PDR195‐mcherry‐*PstHXT1* was co‐transformed with pDR195‐eGFP or pDR195‐*PstCYN1*‐eGFP into the yeast strain ABC733 using the modified lithium acetate transformation method. Transformants were cultured in minimal medium lacking uracil for 48 h. GFP was observed using a confocal laser scanning microscope (Olympus; FV3000).

For subcellular localisation in *N. benthamiana*, the *PstCYN1* CDS was cloned in‐frame upstream of the GFP gene in the pCAMBIA‐1302‐GFP vector to generate the fusion construct *PstCYN1*‐1302‐GFP, and the *PstHXT1* CDS was cloned in‐frame upstream of the mcherry gene in the pich vector to generate the fusion construct *PstHXT1*‐pich‐mcherry. The recombinant constructs and the empty vector control were introduced into *Agrobacterium tumefaciens* GV3101. For transient expression, *Agrobacterium* cultures were suspended in infiltration buffer (10 mM MES, 10 mM MgCl_2_ and 150 μM acetosyringone, pH 5.6) to an OD_600_ of 0.6 and incubated in the dark for 1 h. The suspensions were then co‐infiltrated into *N*. *benthamiana* leaves using a 1 mL needleless syringe. GFP and mcherry fluorescence were examined 36 h post‐infiltration using a confocal laser scanning microscope (Olympus; FV3000). Primer sequences are listed in Table S1.

### 4.6 BSMV‐Induced Gene Silencing

Gene silencing of *PstCYN1* was performed using BSMV‐induced gene silencing (VIGS) as previously described (Holzberg et al. 2002; Scofield et al. 2005). A specific gene fragment of *PstCYN1* was cloned into the BSMV vector to generate the silencing construct BSMV:*PstCYN1*. The constructs BSMV:γ (empty vector) and BSMV:*TaPDS* (phytoene desaturase, positive control) were used as negative and visual controls, respectively. Each BSMV construct was mechanically inoculated onto the second leaves of wheat seedlings.

After inoculation, the seedlings were kept in the dark at 100% relative humidity for 24 h, and then transferred to a growth chamber maintained at 25°C–27°C for 10–12 days. When plants inoculated with BSMV:*TaPDS* displayed the characteristic photobleaching phenotype, the fourth leaves exhibiting viral symptoms were inoculated with freshly harvested urediniospores of Pst race CYR31. Disease symptoms in BSMV‐silenced plants were evaluated at 14 dpi. The experiment was repeated independently three times. Primer sequences used for construct generation are provided in Table S1.

### 4.7 Generation and Identification of Wheat Transgenic Plants

The wheat cultivar Fielder was used to generate transgenic plants. A fragment specific to *PstCYN1* was inserted into the RNAi vector pC336 (Ubi:GWRNAi:Nos) using the Gateway cloning system. The primer pair *PstCYN1*‐RNAi‐F/R (Table S1) was designed based on the selected RNAi fragment's sequence of *PstCYN1*. Stable transformation was performed via *A. tumefaciens* ‐mediated transformation following established protocols. Transgenic plants were screened and confirmed through molecular analysis.

### 4.8 Histological Analyses of Wheat Inoculated With Pst

To visualise fungal structures, infected wheat leaves were stained with wheat germ agglutinin conjugated to Alexa Fluor 488 (WGA‐Alexa 488). The number of infection hyphae (IH), haustoria (H), haustorial mother cells (HMC) and the infection area was examined under a BX‐53 fluorescence microscope and quantified using the cellSens Entry software (Olympus).

For the detection of hydrogen peroxide (H_2_O_2_) accumulation, infected leaves were incubated in a solution of 1 mg/mL 3,3′‐diaminobenzidine (DAB; Coolaber) as described by Duan et al. (2024). Following staining, samples were decolourised in a destaining solution consisting of ethanol and acetic acid (1:1, vol/vol) until chlorophyll was removed and tissues became translucent. Samples were then immersed in chloral hydrate overnight. Observations were made under bright‐field illumination using the Olympus BX‐53 microscope, and the H_2_O_2_‐accumulated area was quantified using cellSens Entry software. At least 30 infection sites were analysed per time point. The experiment was independently repeated three times.

Cell death at infection sites was assessed using trypan blue staining. Inoculated leaves were excised and cut into segments of approximately equal length. Segments were stained in 0.5% trypan blue prepared in lactophenol for 2 days at room temperature and subsequently mounted in 2.5 mg/mL chloral hydrate solution. Stained tissues were observed and imaged using an epifluorescence microscope (Olympus). Thirty infection sites were randomly selected and quantified for each biological replicate. Three independent replicates were performed.

### 4.9 Quantification of Apoplatic Cystine Concentration

The concentration of apoplastic cystine was determined using intercellular washing fluid (IWF), extracted as previously described (Zhang et al. 2025). To quantify free cysteine, thiols were extracted and analysed by reverse‐phase high‐performance liquid chromatography (HPLC). The quantification of L‐cysteine followed the methods of Dominguez‐Solís et al. (2001) and Minocha et al. (2008), with slight modifications. Fresh leaf tissues were collected into 2 mL microcentrifuge tubes and weighed using an analytical balance. Samples were homogenised using a TissueLyser in the presence of 0.1 M HCl containing 1 mM EDTA. The homogenate was centrifuged at 12,000 *g* for 15 min at 4°C, and the supernatant was transferred to a new 1.5 mL microcentrifuge tube. For free cysteine quantification, 330 μL of the supernatant was mixed with 20 μL of 15 mM monobromobimane (mBBr) and incubated in the dark at 45°C for 15 min to derivatise thiol groups. The reaction was stopped by adding 250 μL of 0.25% methanesulphonic acid. To determine total cysteine content, thiols were first reduced with *N*‐cyclohexyltaurine (CHES) and NaBH_4_ at 45°C for 10 min, followed by derivatisation with mBBr as described above.

Derivatised samples (10 μL) were analysed using an Agilent 1260 Infinity II HPLC system equipped with a reverse‐phase C18 column (Diamonsil, 150 mm × 4.6 mm, 5 μm). Solvent A consisted of 10% methanol and 0.25% acetic acid; Solvent B consisted of 90% methanol and 0.25% acetic acid. Both solvents were adjusted to pH 6.5 with 50% NaOH. Thiols were separated using the following gradient: 0–10 min, 8% B; 10–20 min, 8%–40% B (linear); 20–25 min, 40% B; 25–30 min, 40%–90% B (linear); 30–32 min, 90%–100% B; 32–42 min, 8% B. The flow rate was 1.0 mL/min, and the column temperature was maintained at 30°C. Fluorescent derivatives were detected using a fluorescence detector set to an excitation wavelength of 260 nm and an emission wavelength of 474 nm.

A standard curve was generated by plotting the known concentrations (μM) of L‐cysteine standards (*y*‐axis) against the corresponding peak areas (*x*‐axis), and a trendline was fitted to calculate sample concentrations. Cystine concentration was calculated by subtracting the concentration of free cysteine from that of total cysteine.

## Author Contributions

Conceptualisation: Jing Zhao, Zhensheng Kang, Wanlu Duan. Investigation: Wanlu Duan, Yanfei Zhang, Shuohui Yang, Sifan Chen, Jiawen Yuan, Chaoran Zhang. Writing – original draft: Wanlu Duan, Jing Zhao. Writing – review and editing: Jing Zhao, Zhensheng Kang. Supervision: Zhensheng Kang. Funding acquisition: Jing Zhao, Zhensheng Kang.

## Conflicts of Interest

The authors declare no conflicts of interest.

## Supporting information


**Figure S1:** Cysteine transport activity analysis of candidate CgCYN1 homologues in *Pst*.

**Figure S2:** Prediction of transmembrane domain and three‐dimension structure of PstCYN1.

**Figure S3:** Subcellular localisation of PstCYN1 in *Nicotiana benthamiana*.

**Figure S4:** Virus‐induced gene silencing (VIGS) of *PstCYN1* attenuates the virulence of *Puccinia striiformis* f. sp. *tritici* (*Pst*) on wheat.

**Figure S5:** Molecular identification of positive transgenic *PstCYN1*‐RNAi wheat lines.

**Table S1:** Primer list used in this study.

## Data Availability

The data that supports the findings of this study is contained within the article or Supporting Information.
