# Supplementary material for: A Cystine Transporter Mediates Nutrient Acquisition and Redox Balance During Wheat Stripe Rust Infection
Source: Mol Plant Pathol. 2025 Nov 12;26(11):e70172. doi: 10.1111/mpp.70172 (PMC12612559; doi:10.1111/mpp.70172)
Supplement: Supplementary file 1 — Figure S1: Cysteine transport activity analysis of candidate CgCYN1 homologues in Pst. [file MPP-26-e70172-s005.pdf]

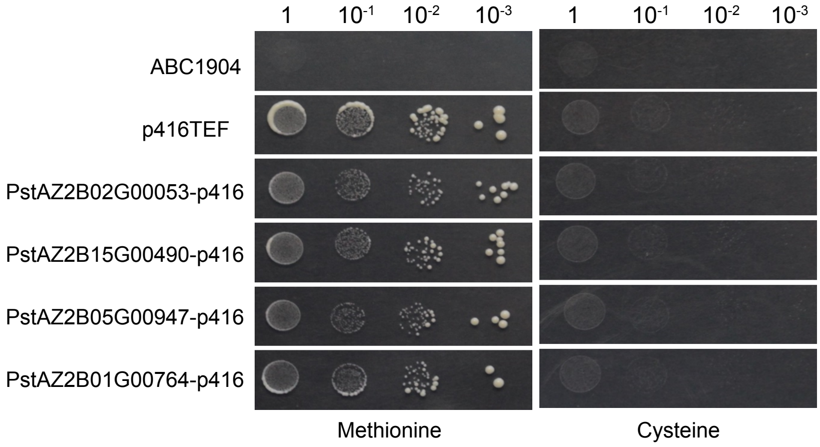

Figure S1. Cysteine transport activity analysis of candidate CgCYN1 homologs in *Pst*.

Growth assay of *Saccharomyces cerevisiae met15Δ* strain ABC1904 transformed with *Pst* candidate homologs of *CgCYN1* or the empty vector control (p416TEF). Yeast transformants were cultured on minimal medium supplemented with either 200 µM cysteine or 200 µM methionine to evaluate cysteine uptake capacity.
