# Supplementary material for: A Cystine Transporter Mediates Nutrient Acquisition and Redox Balance During Wheat Stripe Rust Infection
Source: Mol Plant Pathol. 2025 Nov 12;26(11):e70172. doi: 10.1111/mpp.70172 (PMC12612559; doi:10.1111/mpp.70172)
Supplement: Supplementary file 2 — Figure S2: Prediction of transmembrane domain and three‐dimension structure of PstCYN1. [file MPP-26-e70172-s004.pdf]

a

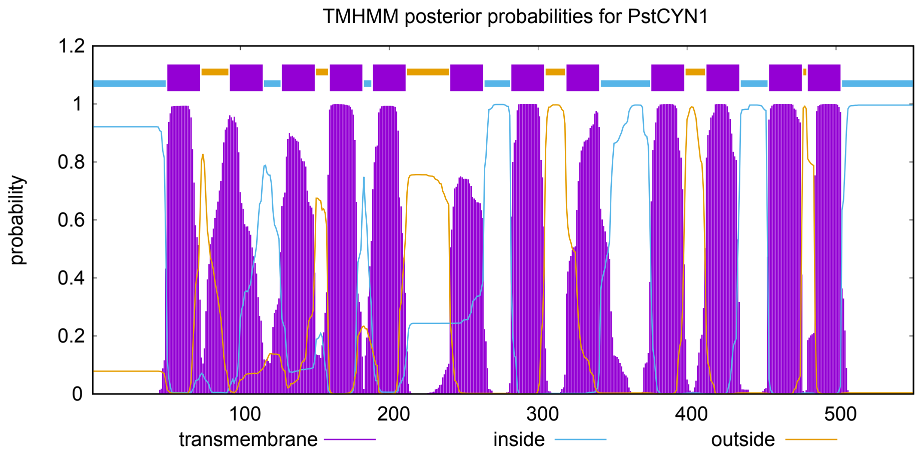

b

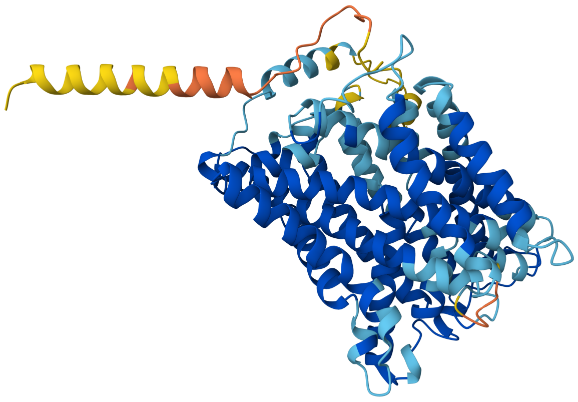

Figure S2. Prediction of transmembrane domain and three-dimension structure of PstCYN1.

(a) Prediction of twelve transmembrane helices in the PstCYN1 protein using TMHMM 2.0, indicating its putative membrane-spanning regions. (b) Three-dimensional structural model of PstCYN1 generated by AlphaFold 3, illustrating the spatial organization of transmembrane domains and overall protein architecture.
