# Supplementary material for: A Cystine Transporter Mediates Nutrient Acquisition and Redox Balance During Wheat Stripe Rust Infection
Source: Mol Plant Pathol. 2025 Nov 12;26(11):e70172. doi: 10.1111/mpp.70172 (PMC12612559; doi:10.1111/mpp.70172)
Supplement: Supplementary file 3 — Figure S3: Subcellular localisation of PstCYN1 in Nicotiana benthamiana. [file MPP-26-e70172-s001.pdf]

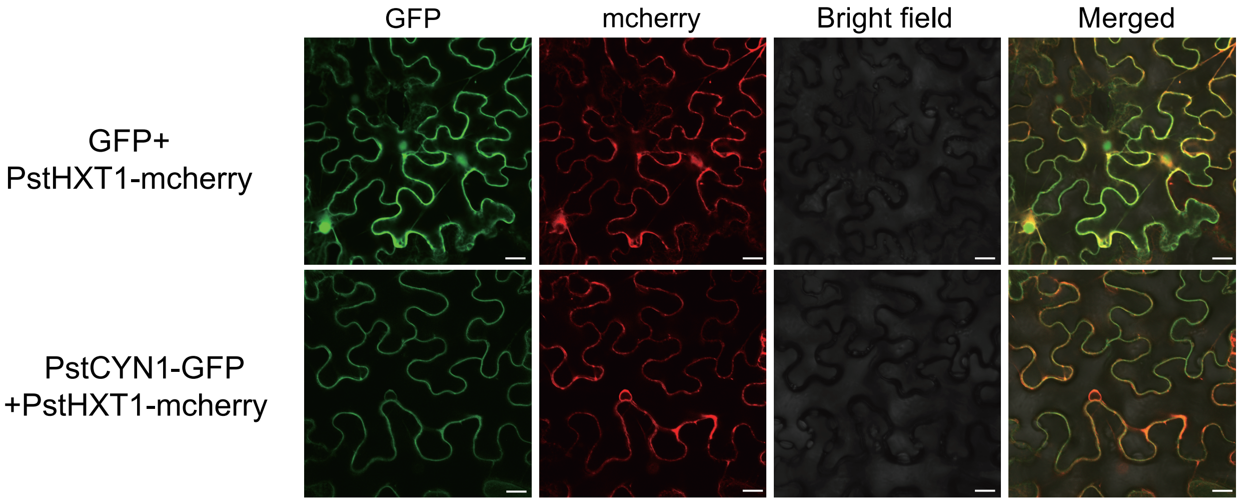

Figure S3. Subcellular localization of PstCYN1 in *Nicotiana benthamiana.*

GFP-tagged PstCYN1 was transiently expressed in *N. benthamiana* leaf cells via *Agrobacterium tumefaciens*–mediated infiltration. PstHXT1-mcherry was used as a plasma membrane marker. GFP and mcherry fluorescence was observed and imaged 2 days post-infiltration using fluorescence microscopy. Scale bars: 20 µm.
