# Supplementary material for: A Cystine Transporter Mediates Nutrient Acquisition and Redox Balance During Wheat Stripe Rust Infection
Source: Mol Plant Pathol. 2025 Nov 12;26(11):e70172. doi: 10.1111/mpp.70172 (PMC12612559; doi:10.1111/mpp.70172)
Supplement: Supplementary file 4 — Figure S4: Virus‐induced gene silencing (VIGS) of PstCYN1 attenuates the virulence of Puccinia striiformis f. sp. tritici (Pst) on wheat. [file MPP-26-e70172-s006.pdf]

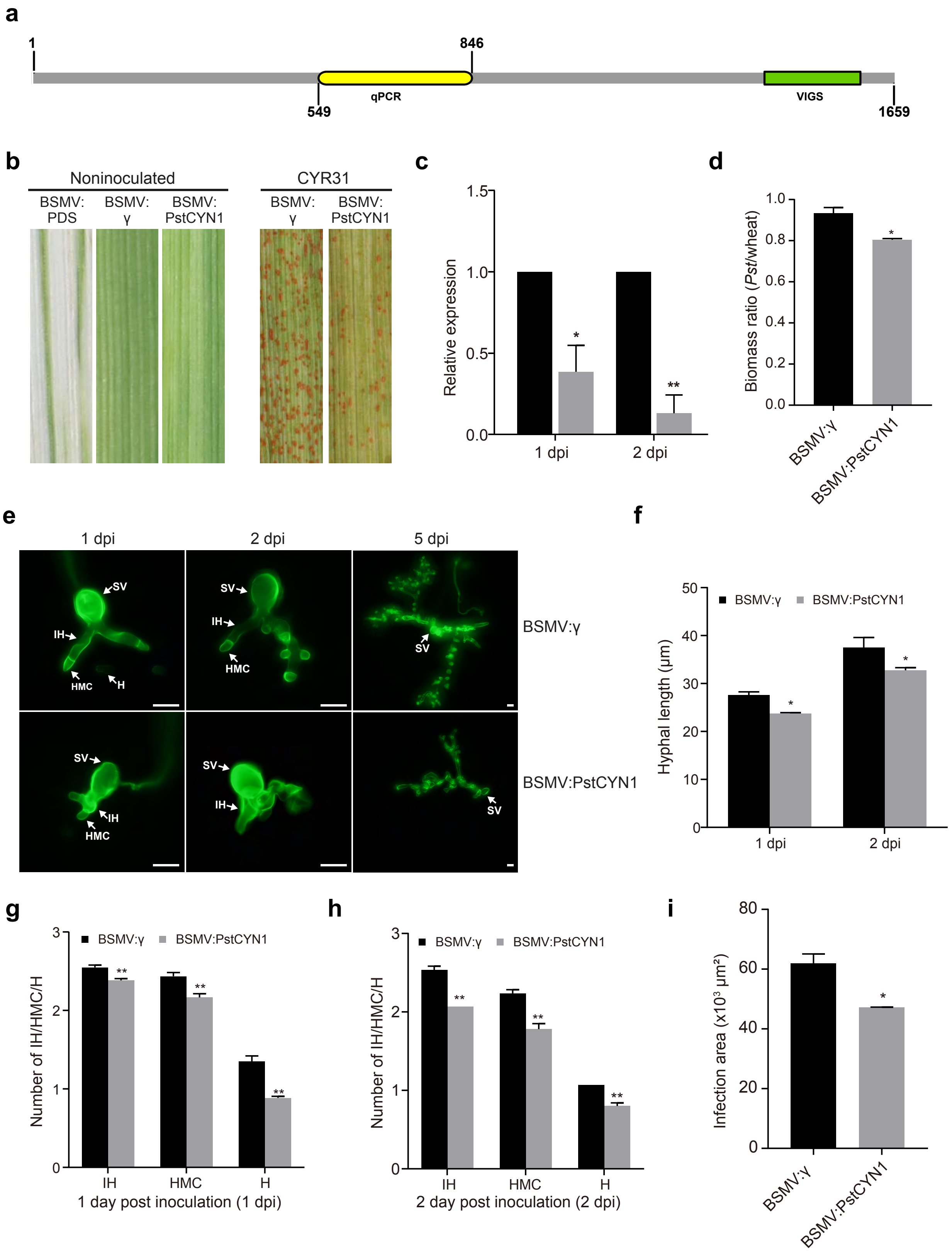

Figure S4. Virus-induced gene silencing (VIGS) of *PstCYN1* attenuates the virulence of *Puccinia striiformis* f. sp. *tritici* (*Pst*) on wheat.

(a) Schematic representation of the *PstCYN1* regions targeted for silencing and qRT-PCR quantification.
(b) Disease phenotype of *PstCYN1*-silenced wheat plants. Second leaves of two-leaf stage wheat cultivar Suwon 11 were inoculated with barley stripe mosaic virus (BSMV:γ or recombinant BSMV:asPstCYN1). Twelve days later, fourth leaves displaying viral symptoms (left) were challenged with *Pst* race CYR31. BSMV:*TaPDS*, exhibiting a photo-bleaching phenotype, was used as a visual silencing control. Disease symptoms were photographed at 14 days post inoculation (dpi) with *Pst* (right).
(c) Transcript levels of *PstCYN1* in *Pst*-infected wheat leaves at 1 and 2 dpi, measured by qRT-PCR and normalized to *PstEF1*. Expression in BSMV:γ was set to 1.
(d) Quantification of fungal biomass at 10 dpi based on the ratio of fungal to host DNA. Total DNA was extracted and normalized using *TaEF-1α* (wheat) and *PstEF1* (*Pst*).
(e) Microscopic observation of *Pst* infection structures at 2 and 5 dpi in *PstCYN1*-silenced leaves. Samples were stained with WGA-488 and examined under fluorescence microscopy. SV, substomatal vesicle; HMC, haustorial mother cell; IH, infection hypha. Scale bars: 20 μm.
(f) Measurement of *Pst* hyphal length at 1 and 2 dpi.
(g-h) Quantification of infection structures (IH, HMC, and H) per site at 1 dpi (g) and 2 dpi (h).
(i) Infection site area at 5 dpi (in μm^2). All values are means ± SD from three independent biological replicates (n = 30 infection sites per replicate). Statistical significance was determined using a two-sided Student's *t*-test (*$P < 0.05$).
