# Supplementary material for: A Cystine Transporter Mediates Nutrient Acquisition and Redox Balance During Wheat Stripe Rust Infection
Source: Mol Plant Pathol. 2025 Nov 12;26(11):e70172. doi: 10.1111/mpp.70172 (PMC12612559; doi:10.1111/mpp.70172)
Supplement: Supplementary file 5 — Figure S5: Molecular identification of positive transgenic PstCYN1‐RNAi wheat lines. [file MPP-26-e70172-s002.pdf]

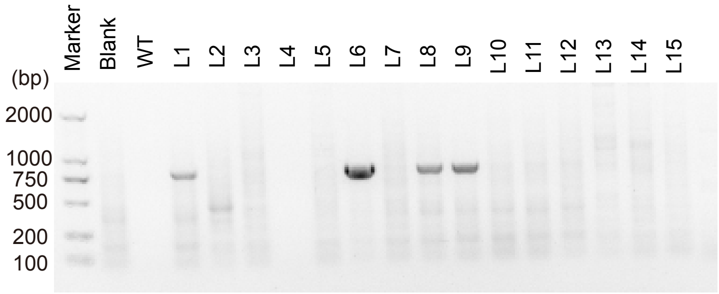

Figure S5. Molecular identification of positive transgenic *PstCYN1*-RNAi wheat lines.

PCR-based screening of wheat plants to identify positive *PstCYN1*-RNAi transgenic lines. Genomic DNA extracted from putative transformants was used as template. DNA from non-transformed Fielder (wild type, WT) was included as a negative control.
